# The clinical and prognostic implication of deep stromal invasion in cervical cancer patients undergoing radical hysterectomy

**DOI:** 10.7150/jca.50752

**Published:** 2020-10-23

**Authors:** Jun Zhu, Lijie Cao, Hao Wen, Rui Bi, Xiaohua Wu, Guihao Ke

**Affiliations:** 1 Department of Oncology, Shanghai Medical College, Fudan University, 130 Dong-An Road, Shanghai 200032, China.; 2 Department of Gynecologic Oncology, Fudan University Shanghai Cancer Center, 270 Dong-An Road, Shanghai 200032, China.; 3 Department of Pathology, Fudan University Shanghai Cancer Center, 270 Dong-An Road, Shanghai 200032, China.

**Keywords:** cervical cancer, deep stromal invasion (DSI), full-thickness, disease recurrence, survival

## Abstract

**Background:** To evaluate the patterns of recurrence and survival related to deep stromal invasion (DSI) in cervical cancer patients who underwent the radical surgery.

**Methods:** Patients with International Federation of Gynaecology and Obstetrics (FIGO) 2009 stage IB and IIA and definite pathology-confirmed deep stromal invasion between 03/2006 and 06/2014 were collected. A subcategorization of deep stromal invasion (inner full-thickness, full-thickness and outer full-thickness) were performed. Disease-free survival (DFS) and overall survival (OS) were compared by Kaplan-Meier analysis and independent predictors were identified using Cox regression analysis.

**Results:** A total of 3,298 cervical cancer patients were included. The proportion of patients with outer 1/3 to full-thickness invasion, full-thickness invasion and outer-full-thickness invasion were 60.6%, 33.5% and 5.9%, respectively. Deep stromal invasion strongly correlated with patients' age, stage, menopause status, tumor diameter, lymphovascular space invasion (LVSI), nodal metastasis, parametrial and vaginal involvement, as well as the site of recurrence. However, no connection was found between the DSI and tumor histologic type. Upon further analysis, patients with full- and outer-full-thickness invasion exhibited significantly higher recurrence rates compared to inner full-thickness group. Both DFS and OS was independently associated with the depth of deep stromal invasion. By subgroup analysis, multivariate analysis revealed that only adjuvant radiotherapy was independent risk factors for both DFS and OS in isolated full-thickness invasion patients.

**Conclusions:** This study indicated that the depth of deep stromal invasion is an important prognostic factor in patients with cervical cancer. Patients with full-thickness invasion should receive customized adjuvant treatment.

## Introduction

Cervical cancer is the 4^th^ most common cancer-related death among all the female malignancies worldwide 1. More than half a million women are newly diagnosed annually, and most cases occur in developing and undeveloped countries 2. Each year, approximately a quarter of the new cases globally are diagnosed in China. For cervical cancer patients with International Federation of Gynaecology and Obstetrics (FIGO) stage IB-IIA, whose diseases clinically confined to the cervix and upper vagina, radical hysterectomy with pelvic lymphadenectomy is considered the standard treatment 3, 4. The 5-year disease-free (DFS) survival was reported to be as high as 75% to 95%, which benefited from the presence of specific prognostic variables 5. Some factors were identified before the operation, such as the palpable tumor volume and extension. After surgery, some important pathological variables were classified as the intermediate and high risk factors, such as tumor volume, lymphovascular space invasion (LVSI), deep stromal invasion (DSI), lymph node metastases, parametrial infiltration, surgical margin and/or positive parametrium, and warranted necessary adjuvant therapy 6-8. However, even if some pathological variables were proved to have impact on survival, they are not incorporated in the staging system.

Adjuvant radiotherapy is recommended for the treatment of patients with intermediate or high-risk factors of the cervical cancer according to the National Comprehensive Cancer Network (NCCN) clinical guidelines 9. However, one situation always encountered at clinical practice was that only one of the intermediate risk predictors, such as isolated DSI without positive LVSI and large tumor volume was presented. As middle or deep 1/3 of stromal invasion is considered the intermediate risk factor and parametrial involvement the high one, full-thickness stromal invasion lies conceptually between micro-invasive parametrial involvement and normal DSI. For these full-thickness invasion tumors, there is little evidence has been shown on the clinico-pathological characteristics as well as the prognostic information. Delgado et al. revealed the relationship between the relative risk of recurrence and the depth of stromal invasion, and found the disease-free survival was 94.1% for superficial third, 84.5% for middle third, and 73.6% for deep third invasion, respectively 3. However, the management of tumors with full-thickness invasion is still treated as just middle or deep 1/3 of stromal invasion. Diseases with outer full-thickness invasion but without positive parametrial margin are also regarded as DSI, and no specific additional treatment is performed in these patients. Moon et al. found that post-operative radiotherapy improved pelvic control in patients with an isolated full-thickness stromal invasion 10. Thus, the purpose of the present study was to evaluate the pattern of recurrence and survival related to different postoperative DSI in stages IB and IIA cervical cancer patients who underwent the radical surgery.

## Methods

### Patients and treatment

The medical records of 3,298 patients with FIGO (2009) stage IB - IIA were reviewed, and all the patients had undergone standard abdominal radical hysterectomy and pelvic lymph node dissection in the Department of Gynecologic Oncology, Fudan University Shanghai Cancer Center (China) between 2006 and 2014. The resection of para-aortic lymph node was performed if suspected para-aortic lymph node involvement was found before or during the operation, as well as the intraoperative fast frozen histology confirmed positive para-aortic or standard iliac lymph nodes. One gynecologic pathologist had reviewed each microscopic slide and a second experienced pathologist carried out confirmation. The inclusion criteria of the study were as follows: 1) post-operative histological confirmation of squamous carcinoma, adenocarcinoma and adenosquamous carcinoma; 2) post-operative histological confirmation of deep stromal invasion including middle or deep 1/3 of stromal invasion, full-thickness and outer full-thickness invasion; 3) no preoperative treatment including chemotherapy and radiotherapy; and 4) the compliance with strict follow-up visits in our center. Patients with past cancer history and preoperative diagnostic conization were excluded in this study. The study received approval (approval No.1910208-7) from the Ethics Committee at Fudan University Shanghai Cancer Center, Shanghai, China.

Postoperative adjuvant therapeutic strategies included pelvic external beam radiotherapy (EBRT) and concurrent platinumbased chemotherapy in patients with intermediate- (LVSI, deep stromal invasion and tumor size) and high-risk factors (positive margin, lymph node metastasis and positive parametria) according to the NCCN clinical guidelines. Patients with positive common iliac lymph node or para-aortic lymph received the extended-field EBRT. Systematic intravenous chemotherapy (carboplatin/cisplatin + paclitaxel) was administered to those who had more than two positive lymph nodes after adjuvant radiotherapy. During the routine followed up visits, physical examination, Papanicolau smear, routine blood test, and serum tumor markers were performed every 3 months for the first 2 years, every 6 months for the next three years, and yearly afterward. Additional radiographic examinations were performed if a suspected recurrent disease was detected.

A diagnosis of recurrent lesion was made as new or progressive lesions confirmed by histologic and radiologic examination. Cases were further categorized into two groups according to the region of recurrent lesion: pelvic and extrapelvic recurrence.

### Subcategorization of DSI

Depth of stromal invasion was evaluated after a microscopic examination by an experienced pathologist and was defined as more than 1 mm in depth of stromal, which is routinely accessed in our center. According to the fractions of cervical wall thickness, depth of stromal invasion was expressed as inner third, middle third, outer third, full-thickness and outer full-thickness, which was routinely performed by the histopathologist in our center. If the tumor was middle third infiltration, a specific description of either more than half infiltration or not was used. Full-thickness infiltration was defined as only the whole cervical invasion but no involvement of cervical-parametrial transition zone. Outer full-thickness invasion was used as the microscopic involvement of the cervical-parametrial transition zone without parametrial involvement was found. Thus, outer full-thickness invasion without positive parametrial margin is not regarded as the high-risk factor but only the intermediate risk predictors. Patients in the cohort were categorized into three groups according to the specific depth of stromal invasion: inner full-thickness (IF, middle third depth to full-thickness), full-thickness (FT) and outer full-thickness invasion (OF).

### Statistical analyses

Patients' clinicopathological characteristics and survival were compared using the χ^2^ test or Fisher's Exact Test for frequencies and the Mann-Whitney U test for continuous variables. The probabilities of DFS, OS were calculated using the Kaplan-Meier method, and the log-rank test was further used to compare survival curves. COX proportional hazards models were developed by forward, stepwise regression to identify independent predictors related to recurrence and survival. Statistical significance existed when *P <* 0.05. All analyses were carried out using SPSS software (release 17.0, SPSS Inc, Chicago, IL).

## Results

### Clinical and pathological characteristics

**Table 1** revealed the clinicopathological features of the patients. A total of 3,298 patients with postoperative deep stromal invasion were included. The median age of all patients was 47.0 years (range 19 - 80). The specific FIGO stage of all the patients were listed as follows: IB1 (1,165, 35.3%), IB2 (308, 9.3%), IIA1 (1,252, 38.0%), and IIA2 (573, 17.4%). The majority of histological subtype was squamous cell carcinoma (2,936, 89.0%), and adenocarcinoma and adenosquamous carcinoma were found in 245 (7.4%) and 117 (3.5%) patients, respectively. Approximately 60.6% (1,998/3,298) of the cases presented with outer middle third to full-thickness stromal invasion, 33.5% (1,106/3,298) of the patients were full-thickness stromal invasion, and only 5.9% (194/3,298) patients had outer full-thickness invasion. Overall, positive LVSI, lymph node metastasis, parametrial involvement, and vaginal margin invasion accounted for 44.7, 33.2, 7.4, and 3.2%, respectively. Among the whole cohort, 2,334 (70.8%) patients received adjuvant radiotherapy and 1,137 (34.5%) cases had adjuvant chemotherapy.

### Association between DSI and the clinicopathological features

The association between the DSI and clinicopathological characteristics was shown in **Table 1.** Comparison of between the three groups revealed statistically significant differences in terms of age at diagnosis (*P <* 0.001), FIGO stage (*P <* 0.001), menopause status (*P <* 0.001), tumor diameter (*P <* 0.001), LVSI (*P <* 0.001), lymph node metastasis (*P <* 0.001), parametrial involvement (*P <* 0.001), vaginal margin invasion (*P <* 0.001), recurrent region (*P <* 0.001), and adjuvant treatment (*P <* 0.001). No statistically significant differences were found in histology subtype (*P* = 0.817) and parity (*P* = 0.115). These results indicate that different depth of stromal invasion might present different biological behaviors.

### Survival and patterns of failure

**Figure 1:**
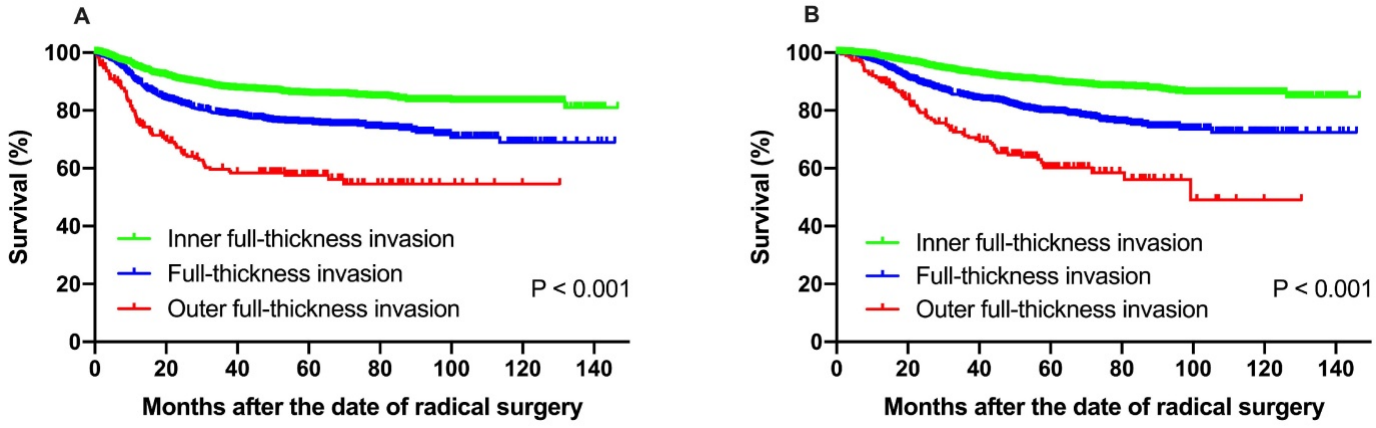
(**A**) Disease-free survival, (**B**) overall survival of patients with IF, FT and OF group. The 5-year DFS at IF, FT and OF group were 85.6%, 75.8% and 57.5%, respectively, according to Kaplan-Meier analysis and 5-year DFS rates in FT and OF group were significantly decreased compared to those in the IF group (*P <* 0.001). The 5-year OS rate for the patients with IF, FT and OF group were 89.9%, 79.5% and 60.2%, respectively, and statistically significant difference was also found (*P <* 0.001).

The median follow-up time (range) of all the patients was 59.1 months (range 1.9-146.6 months). The 5-year DFS and OS rate was 80.7% and 84.7%, respectively. For the entire cohort, 187 (31.0%) patients had pelvic cavity recurrences, 346 (57.4%) patients experienced extrapelvic recurrences, and 70 (11.6%) cases had both pelvic and extrapelvic recurrences. The 601 patients with recurrent diseases had a median survival time of 30.2 months. Statistically significant difference was observed when different depth of stromal invasion was compared. The 5-year DFS at IF, FT and OF group were 85.6%, 75.8% and 57.5%, respectively, according to Kaplan-Meier analysis and 5-year DFS rates in FT and OF group were significantly decreased compared to those in the IF group (*P <* 0.001). The 5-year OS rate for the patients with IF, FT and OF group were 89.9%, 79.5% and 60.2%, respectively, and statistically significant difference was also found (*P <* 0.001) (**Fig. 1**).

### Prognostic factors for DFS and OS

Variables found to be associated with DFS and OS were summarized in **Table 2.** On univariate analysis, histologic types, FIGO stage, depth of stromal invasion, tumor diameter, DSI, LVSI, lymph node metastasis, parametrial involvement, vaginal margin invasion, recurrence region, adjuvant radiotherapy and chemotherapy were significant predictors for both DFS and OS. The Cox regression analysis showed that histologic type, DSI, LVSI, lymph node metastasis, parametrial involvement and vaginal margin invasion were independent prognostic factors for both DFS and OS.

### Clinical and survival analysis in separate histologic subgroups

**Figure 2:**
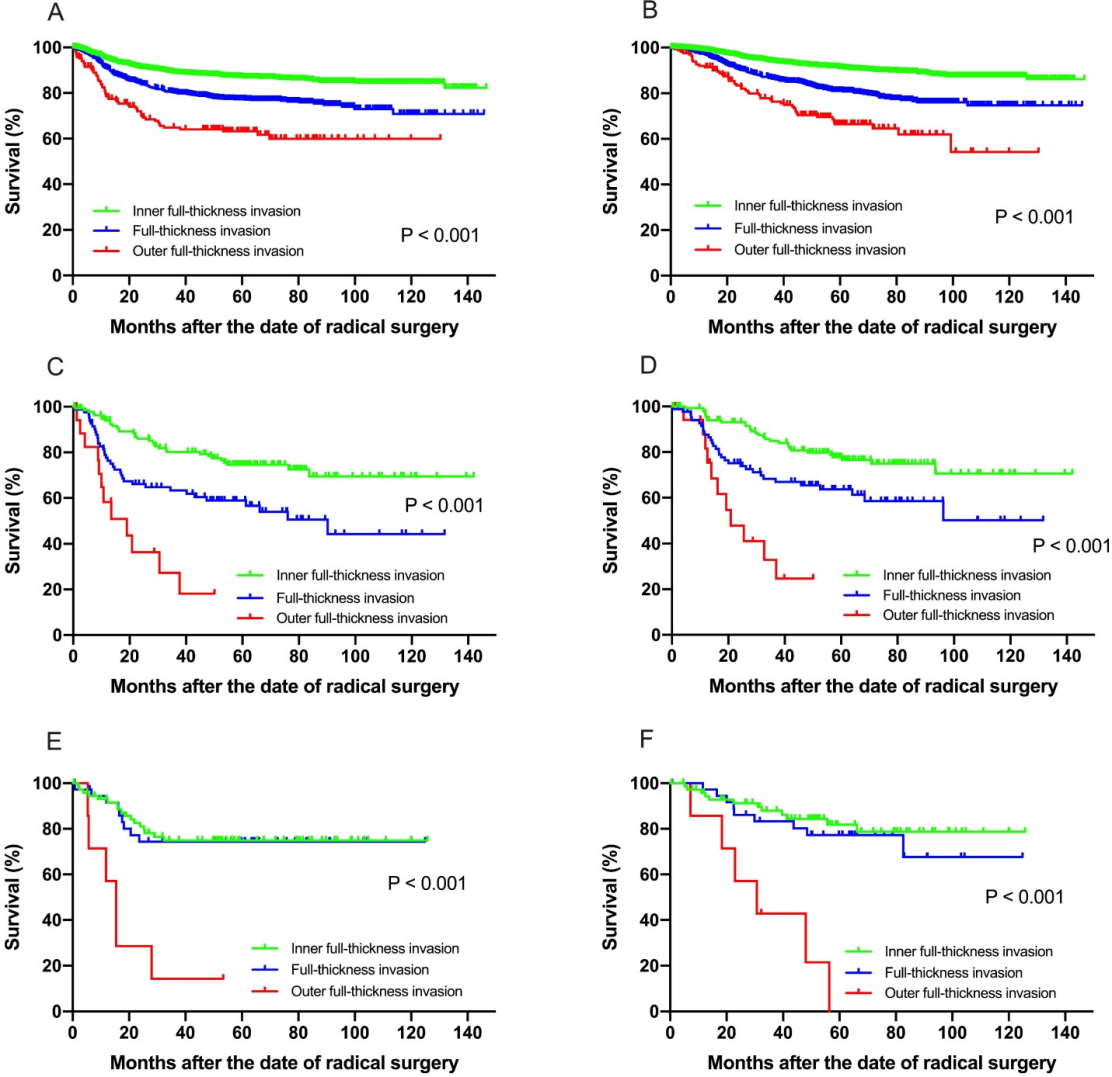
(**A**) Disease-free survival, (**B**) overall survival among patients with squamous cell carcinoma for recurrence and survival. Both the DFS and OS in patients with squamous cell carcinoma show significant difference at IF, FT and OF group (*P <* 0.001). Similarly, the DFS (**C**) and OS (**D**) in patients with adenocarcinoma carcinoma show significant difference at IF, FT and OF group (*P <* 0.001). For patients with adenosquamous carcinoma, the OF group showed significant worse survival for DFS (**E**) and OS (**F**) than the IF and FT group.

Subgroup analysis was then performed in separate histologic type patients for both recurrence and survival (Supplementary Tables 1-3). Among patients with squamous cell carcinoma, multivariate analysis revealed that FIGO stage, depth of stromal invasion, DSI, LVSI, lymph node metastasis, parametrial involvement, vaginal margin invasion were independent prognostic factors for both DFS and OS (**Fig. 2A & B**). In patients with adenocarcinoma, only preoperative serum cancer antigen 125 (CA125) and lymph node metastasis were found to be independent prognostic factors for both DFS and OS (**Fig. 2C & D**). However, only preoperative serum cancer antigen 125 (CA125) and outer full-thickness invasion were found to be independently significant to OS in adenosquamous carcinoma (**Fig. 2E & F**).

### Clinical and survival analysis in isolated inter-mediate risk factors subgroups

In order to avoid the negative effect of high-risk factors on recurrence and survival, subgroup analysis was conducted among patients with isolated inter-mediate risk factors for recurrence and survival. A total of 2,105 patients with one or more inter-mediate risk factors were included (**Table 3**). Univariate analysis revealed that FIGO stage (*P* = 0.023), depth of stromal invasion (*P* = 0.004), LVSI (*P <* 0.001) and recurrence region (*P* = 0.004) were significant predictors for DFS. Histologic type (*P* = 0.011), FIGO stage (*P <* 0.001), menopause status (*P <* 0.001), DSI (*P <* 0.001), tumor diameter (*P* = 0.005), LVSI (*P <* 0.001) and recurrence region (*P* = 0.027) were significant predictors for OS.

**Figure 3:**
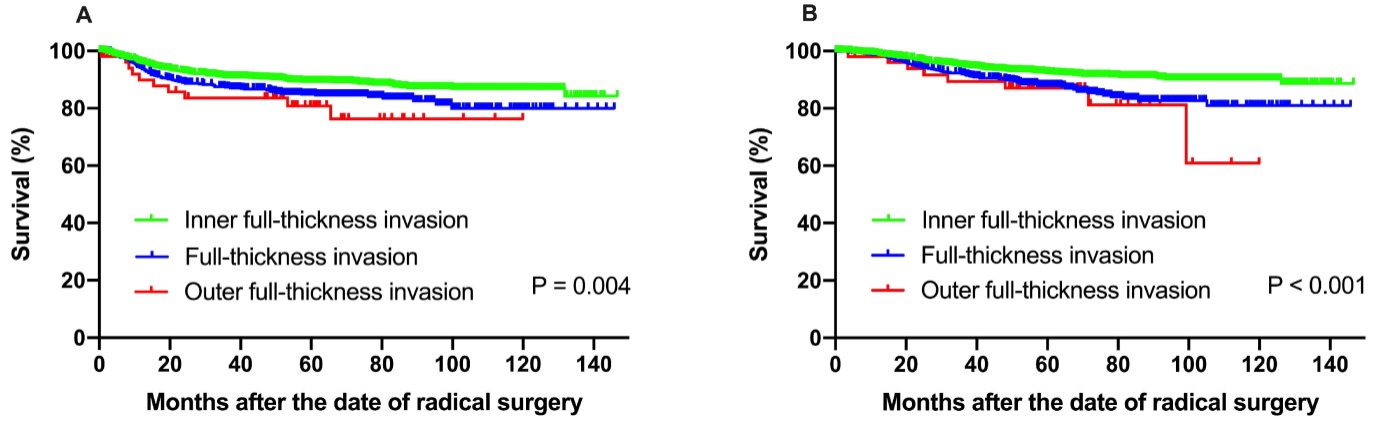
(**A**) Disease-free survival, (**B**) overall survival among patients with isolated inter-mediate risk factors for recurrence and survival. The 5-year DFS at IF, FT and OF group were 89.3%, 84.9% and 80.8%, respectively (*P* = 0.004). The 5-year OS rate for the patients with IF, FT and OF group were 92.4%, 88.0% and 87.1%, respectively, with significant difference (*P <* 0.001).

The 5-year DFS at IF, FT and OF group were 89.3%, 84.9% and 80.8%, respectively (*P* = 0.004). The 5-year OS rate for the patients with IF, FT and OF group were 92.4%, 88.0% and 87.1%, respectively, with significant difference (*P <* 0.001) (**Fig. 3**).

The Cox regression analysis confirmed that adenocarcinoma subtype, FIGO stage, full-thickness invasion, and LVSI were independent prognostic factors for DFS. Histologic subtype, FIGO stage, full-thickness invasion, tumor diamater, and LVSI were found to be independently significant to OS (**Table 4**).

### Clinical and survival analysis in isolated full-thickness invasion

The records of 217 patients with isolated full-thickness invasion without any other unfavorable high and inter-mediate pathological findings were further analyzed. The median age of all patients was 52.1 years (range 24-80). The specific FIGO stage of all the patients were listed as follows: IB1 (63, 29.0%), IB2 (11, 5.1%), IIA1 (109, 50.2%), and IIA2 (34, 15.7%). The majority of histological subtype was squamous cell carcinoma (189, 87.1%), and adenocarcinoma and adenosquamous carcinoma were found in 21 (9.7%) and 7 (3.2%) patients, respectively.

With a median follow-up of 63.1 months (range 8.1-143.5 months), univariate analysis showed that adjuvant radiotherapy was significantly prognostic for both DFS (*P* = 0.009) and OS (*P* = 0.029) (**Fig. 2**). Multivariate analysis revealed that only adjuvant radiotherapy was independent risk factors for both DFS (hazard ratio [HR] = 0.311, 95% CI, 0.098 - 0.983,* P* = 0.047) and OS (HR = 0.221, 95% CI, 0.059 - 0.834, *P* = 0.026) in isolated full-thickness invasion cases (**Table 5**).

## Discussion

This study aimed to evaluate the different patterns of recurrence and survival related to subcategorization of DSI in cervical cancer patients who underwent the radical surgery, and to explore the optimal management for patients with ≥ full-thickness invasion after radical hysterectomy in stages IB - IIA cervical carcinoma.

Cervical cancer patients with some postoperative pathological findings of high-risk factors for recurrence are warranted necessary adjuvant treatment, which including positive lymph nodes, surgical margin and/or parametrial invasion 7, 11. In addition, most majority of patients with early stage tumor present one or more intermediate-risk factors, including great tumor volume, LVSI, or DSI, which are closely related to recurrence. These patients are often advised to receive adjuvant pelvic radiotherapy. Deep stromal invasion, defined as the fractions of cervical wall thickness, is one of the intermediate risk factors often encountered in clinical settings. However, the criteria of depth of invasion is only expressed as inner third, middle third, and outer third of cervical wall thickness according to the Sedlis criteria 12. Moreover, full-thickness or outer full-thickness invasion is one frequently encountered situation at clinical practice.

Unlike obvious parametrial involvement, tumors with full-thickness infiltration only invade the whole cervical wall but not reaching the cervical-parametrial transition zone. Besides, situations with outer full-thickness invasion were observed as the microscopic involvement of the cervical-parametrial transition zone without parametrial involvement. It brings the question if full-thickness and outer full-thickness invasion without positive parametrial margins should be treated the same as DSI. This microinvasion can only be diagnosed under microscope, and it is easy to be neglected by clinical examination, resulting in more patients with ≥ full-thickness stromal invasion receiving surgical treatment. Little evidence has been presented on the difference of clinical and prognostic characteristics between full-thickness invasion and DSI, or whether full-thickness should be regarded as a major or minor risk factor. In this study we compared the relationship with clinicopathological characteristics of patients with different DSI. In the present study, patients were analyzed according to the depth of deep stromal invasion, tumors with OF and FT differs from those with IF in those were associated with high prevalence of great tumor burden, positive LVSI, lymph nodes, parametrium and surgical margin. Full-thickness invasion seemed to be more aggressive than inner full-thickness invasive tumors.

It has been reported that deep cervical invasion was strongly associated with a parametrial disease 13, 14. Our findings in conjunction with the studies above clearly support the hypothesis that the tumor is getting close to spread to parametrium with the depth of invasion increases. Pathologically, it might be hypothesized that once the tumor spreads out of the whole cervical wall, the peritoneal layer covering the surface of the cervix is easily to be invaded. However, this pathologic risk factors could not be evaluated before surgery. Usually, invasive lesions in cervical cancer patients who presented visible lesions can be detected by colposcopy-guided biopsy methods. However, it was not possible for the physicians to collect enough tissues of specimens to evaluate the detailed depth of stromal invasion. Moreover, cervical biopsy and excision procedure specimens lack sufficient predictive value for the depth of stromal invasion in the final surgical specimens of cervical cancer patients 15. Therefore, it seems reasonable that patients with full-thickness and even outer full-thickness invasion should be managed with a differential treatment, rather than the same treatment modality as middle third or outer third of stromal invasion diseases.

Previous studies have revealed that DSI was not only closely related to cervical cancer recurrence, but also an independent factor for poor survival 3, 8. Our findings were in accord with their results that depth of stromal invasion was an independently prognostic factor for both DFS and OS. The different depth of deep stromal invasion exhibited significant difference of prognosis. A Korean retrospective study reported the recurrent risk had a positive relationship with the depth of stromal invasion 16. Another GOG study reported an DFS of 73% to 85% in patients with an isolated middle or deep third stromal invasion 3, which is in accordance with our results. However, patients with isolated full-thickness and outer full-thickness invasion had relatively decreased survival rate, and this strengthened the idea that full-thickness and even outer full-thickness invasion might have higher risk of recurrence than the inner full-thickness invasion.

The relative risk of the depth of stromal invasion on recurrence and survival was found to be proportional to postoperative adjuvant therapy 4, 16, 17. In a retrospective study, Li et al. compared the DFS of postoperative adjuvant chemotherapy and adjuvant radiation in patients with DSI. Their results showed that patients exhibited inferior responses to chemotherapy compared to radiotherapy, and patients with full-thickness invasion had increased risk of recurrence 18. Moon et al. evaluated the potential benefit of postoperative radiotherapy in patients with isolated full-thickness cervical stromal invasion in FIGO stages IB - IIA cervical carcinoma patients, and found that compared to having no adjuvant treatment, postoperative radiotherapy could bring increased DFS and pelvic-failure-free survival 10. However, in a study reported by Shimada et al. revealed that patients with isolated DSI who received no PORT experienced no recurrence. For this reason, they suggested that isolated DSI cervical cancer patients should not be used for adjuvant radiotherapy and that DSI was not a prognostic predictor 19. Actually, the definition of DSI in their study was less than 3 mm from the serosa, other than the definition used in most of studies. The clinicopathological results of the DSI patients were also not described in the study. In the present study, postoperative radiotherapy was shown to be the independently prognostic factor for survival. In the meantime, our results also suggested that extrapelvic recurrence occurred in most patients with full-thickness invasion after radical surgery. It seemed that postoperative radiotherapy in patients with full-thickness invasion might reduce recurrence and improve survival, and patients with full-thickness invasion might be the target population for the adjuvant radiation.

Despite the retrospective nature of the present study, a major strength of our study lies in that it is the largest study to date, particularly regarding DSI patients with cervical carcinoma, which could scarcely be available by prospective randomized controlled trials. The detailed DSI can be only reached by surgical results. The evaluation of cervical carcinoma with full-thickness stromal invasion by MRI examination was reported to be limited 20. Since full-thickness invasion remains a common event in resectable cervical cancer, optimal treatment is eagerly awaited to improve the prognosis.

## Conclusion

In conclusion, we have demonstrated that the DSI is an important prognostic factor in patients with cervical cancer. For patients with isolated full-thickness invasion without any other unfavorable pathological findings, adjuvant postoperative radiotherapy should be administered. We believe that the assessment of the status of full-thickness and outer full-thickness stromal invasion without positive parametrial margins is important in formulating the individualized management.

## Supplementary Material

Supplementary tables.

## Figures and Tables

**Table 1:** Clinical and pathologic characteristics according to the depth of stromal invasion groups

Variable	Patients (%)	Subcategorization of DSI			*P* value
	Total = 3298	<full-thickness (n=1998, 60.6%)	full-thickness (n=1106, 33.5%)	> full-thickness (n=194, 5.9%)	
Median age (years)	47.0 (ranging 19 - 80)	46.0 (ranging 19 - 79)	49.0 (ranging 20 - 78)	52.0 (ranging 20 - 80)	< 0.001
**Histologic type**					0.817
Squamous carcinoma	2936 (89.0%)	1783 (89.2%)	984 (89.0%)	169 (87.1%)	
Adenocarcinoma	245 (7.4%)	142 (7.1%)	85 (7.7%)	18 (9.3%)	
Adenosquamous carcinoma	117 (3.5%)	73 (3.7%)	37 (3.3%)	7 (3.6%)	
**FIGO stage (2009)**					< 0.001
IB1	1165 (35.3%)	881 (44.1%)	258 (23.3%)	26 (13.4%)	
IB2	308 (9.3%)	193 (9.7%)	100 (9.0%)	15 (7.7%)	
IIA1	1252 (38.0%)	680 (34.0%)	468 (42.3%)	104 (53.6%)	
IIA2	573 (17.4%)	244 (12.2%)	280 (25.3%)	49 (25.3%)	
**Menopause status**					< 0.001
Menopause	1136 (34.4%)	616 (30.8%)	429 (38.8%)	91 (46.9%)	
Premenopause	2162 (65.6%)	1382 (69.2%)	677 (61.2%)	103 (53.1%)	
**Parity**					0.115
Yes	3150 (95.5%)	1901 (95.1%)	1067 (96.5%)	182 (93.8%)	
No	148 (4.5%)	97 (4.9%)	39 (3.5%)	12 (6.2%)	
Mean tumor diameter (cm)	3.9 (ranging 0.4 - 12.0)	3.7 (ranging 0.4 - 12.0)	4.3 (ranging 1.0 - 11.0)	4.3 (ranging 1.1 - 11.0)	< 0.001
**Lymphovascular space invasion**					< 0.001
Yes	1473 (44.7%)	761 (38.1%)	578 (52.3%)	134 (69.1%)	
No	1825 (55.3%)	1237 (61.9%)	528 (47.7%)	60 (30.9%)	
**Lymph node metastasis**					< 0.001
Yes	1060 (33.2%)	469 (24.5%)	465 (42.8%)	126 (66.3%)	
Only Pelvic	962	440	418	104	
Only Para-aortic	4	3	1	0	
Pelvic + Para-aortic	71	22	33	16	
No	2128 (66.8%)	1443 (75.5%)	621 (57.2%)	64 (33.7%)	
**Parametrial involvement**					< 0.001
Yes	244 (7.4%)	91 (4.6%)	105 (9.5%)	48 (24.7%)	
No	3054 (92.6%)	1907 (95.4%)	1001 (90.5%)	146 (75.3%)	
**Vaginal margin invasion**					< 0.001
Yes	106 (3.2%)	48 (2.4%)	43 (3.9%)	15 (7.7%)	
No	3192 (96.8%)	1950 (97.6%)	1063 (96.1%)	179 (92.3%)	
**Recurrent region**					0.029
Only pelvic	187 (31.0%)	99 (36.5%)	69 (27.1%)	19 (24.7%)	
Only extrapelvic	346 (57.4%)	142 (52.4%)	160 (62.7%)	44 (57.1%)	
Pelvic + extrapelvic	70 (11.6%)	30 (11.1%)	26 (10.2%)	14 (18.2%)	
**Adjuvant radiotherapy**					< 0.001
Yes	2334 (70.8%)	1320 (66.1%)	860 (77.8%)	154 (79.4%)	
No	340 (10.3%)	231 (11.6%)	100 (9.0%)	9 (4.6%)	
Unknown	624 (18.9%)	447 (22.4%)	146 (13.2%)	31 (16.0%)	
**Adjuvant chemotherapy**					< 0.001
Yes	1137 (34.5%)	632 (31.6%)	421 (38.1%)	84 (43.3%)	
No	1399 (42.4%)	843 (42.2%)	492 (44.5%)	64 (33.0%)	
Unknown	762 (23.1%)	523 (26.2%)	193 (17.5%)	46 (23.7%)	

**Table 2:** Univariate and multivariate analysis of the predictors for disease-free survival (DFS) and overall survival (OS) among the whole patients

Variable	DFS				OS			
	Univariate	Multivariate			Univariate	Multivariate		
	*P* value	HR	95% CI	*P* value	*P* value	HR	95% CI	*P* value
**Histologic type**	< 0.001				< 0.001			
Squamous carcinoma		Ref				Ref		
Adenocarcinoma		2.470	1.941 - 3.144	< 0.001		3.044	2.352 - 3.941	< 0.001
Adenosquamous carcinoma		1.715	1.198 - 2.454	0.003		1.76	1.190 - 2.604	0.005
**FIGO stage (2009)**	< 0.001	1.243	1.039 - 1.487	0.018	< 0.001	1.448	1.179 - 1.777	< 0.001
IB								
IIA								
**Menopause status**	0.161				< 0.001			
Menopause								
Premenopause								
**Parity**	0.952				0.650			
Yes								
No								
**Subcategorization of DSI**	< 0.001				< 0.001			
< full-thickness		Ref				Ref		
full-thickness		1.374	1.145 - 1.649	0.001		1.527	1.244 - 1.874	< 0.001
> full-thickness		2.084	1.580 - 2.749	< 0.001		2.281	1.688 - 3.082	< 0.001
**Tumor diameter (cm)**	< 0.001	1.175	0.991 - 1.394	0.064	< 0.001	1.273	1.054 - 1.537	0.012
≤ 4								
> 4								
**Lymphovascular space invasion**	< 0.001	1.874	1.544 - 2.274	< 0.001	< 0.001	2.012	1.614 - 2.508	< 0.001
Yes								
No								
**Lymph node metastasis**	< 0.001	1.758	1.455 - 2.125	< 0.001	< 0.001	1.683	1.360 - 2.083	< 0.001
Yes								
No								
**Parametrial involvement**	< 0.001	1.536	1.212 - 1.949	< 0.001	< 0.001	1.64	1.274 - 2.111	< 0.001
Yes								
No								
**Vaginal margin invasion**	< 0.001	1.829	1.307 - 2.560	< 0.001	< 0.001	1.728	1.198 - 2.492	0.003
Yes								
No								
**Recurrence region**	0.010				0.004			
Only pelvic								
Only extrapelvic								
Pelvic + extrapelvic								
**Adjuvant radiotherapy**	0.046				0.022			
Yes								
No								
**Adjuvant chemotherapy**	< 0.001				< 0.001			
Yes								

**Table 3:** Multivariate analysis of the predictors for DFS and OS among patients with isolated full-thickness invasion without any other unfavorable pathological findings

Variable	DFS			OS		
	Multivariate			Multivariate		
	HR	95% CI	*P* value	HR	95% CI	*P* value
**Histologic type**	1.300	0.514 - 3.283	0.579	1.139	0.313 - 4.147	0.843
Squamous carcinoma						
Adenocarcinoma						
Adenosquamous carcinoma						
**FIGO stage (2009)**	1.962	0.683 - 5.638	0.211	2.195	0.594 - 8.118	0.239
IB						
IIA						
**Adjuvant radiotherapy**	0.311	0.098 - 0.983	0.047	0.221	0.059 - 0.834	0.026
Yes						
No						
**Adjuvant chemotherapy**	0.898	0.280 - 2.884	0.856	0.521	0.133 - 2.040	0.350
Yes						
No						

**Table 4:** Clinical and pathologic characteristics according to the depth of stromal invasion groups among patients with isolated inter-mediate risk factors

Variable	Patients (%)	Subcategorization of DSI			*P* value
	Total = 2105	< full-thickness (n=1459, 69.3%)	full-thickness (n=594, 28.2%)	> full-thickness (n=52, 2.5%)	
Mean age (years)	47.8 (ranging 19 - 80)	46.8 (ranging 19 - 80)	49.9 (ranging 23 - 79)	51.6 (ranging 33 - 74)	< 0.001
**Histologic type**					0.291
Squamous carcinoma	1906 (90.5%)	1314 (90.1%)	541 (91.1%)	51 (98.1%)	
Adenocarcinoma	130 (6.2%)	93 (6.2%)	37 (6.2%)	0 (0)	
Adenosquamous carcinoma	69 (3.3%)	52 (3.6%)	16 (2.7%)	1 (1.9%)	
**FIGO stage (2009)**					< 0.001
IB1	851 (40.4%)	688 (47.2%)	154 (25.9%)	9 (17.3%)	
IB2	184 (8.7%)	129 (8.8%)	52 (8.8%)	3 (5.8%)	
IIA1	760 (36.1%)	478 (32.8%)	253 (42.6%)	29 (55.8%)	
IIA2	310 (14.7%)	164 (11.2%)	135 (22.7%)	11 (21.2%)	
**Menopause status**					< 0.001
Menopause	732 (34.8%)	451 (30.9%)	253 (42.6%)	28 (53.8%)	
Premenopause	1373 (65.2%)	1008 (69.1%)	341 (57.4%)	24 (46.2%)	
**Parity**					0.175
Yes	87 (4.1%)	68 (4.7%)	18 (3.0%)	1 (1.9%)	
No	2018 (95.9%)	1391 (95.3%)	576 (97.0%)	51 (98.1%)	
Mean tumor diameter (cm)	3.8 (ranging 0.4 - 12.0)	3.6 (ranging 0.4 - 12.0)	4.3 (ranging 1.4 - 11.0)	3.9 (ranging 1.1 - 6.5)	0.023
**Lymphovascular space invasion**					0.034
Yes	611 (29.0%)	402 (27.6%)	188 (31.6%)	21 (40.4%)	
No	1494 (71.0%)	1057 (72.4%)	406 (68.4%)	31 (59.6%)	
**Recurrent region**					0.454
Only pelvic	95 (38.0%)	62 (41.1%)	28 (31.8%)	5 (45.5%)	
Only extrapelvic	134 (53.6%)	75 (49.7%)	53 (60.2%)	6 (54.5%)	
Pelvic + extrapelvic	21 (8.4%)	14 (9.3%)	7 (8.0%)	0 (0)	

**Table 5:** Univariate and multivariate analysis of the predictors for DFS and OS among patients with isolated inter-mediate risk factors

Variable	DFS				OS			
	Univariate	Multivariate			Univariate	Multivariate		
	*P* value	HR	95% CI	*P* value	*P* value	HR	95% CI	*P* value
**Histologic type**	0.083				0.011			
Squamous carcinoma		Ref				Ref		
Adenocarcinoma		1.746	1.131 - 2.696	0.012		2.187	1.350 - 3.545	0.001
Adenosquamous carcinoma		1.446	0.787 - 2.656	0.234		2.140	1.157 - 3.959	0.015
**FIGO stage (2009)**	0.023	1.342	1.035 - 1.741	0.026	< 0.001	1.663	1.229 - 2.250	0.001
IB								
IIA								
**Menopause status**	0.053				< 0.001			
Menopause								
Premenopause								
**Parity**	0.947				0.939			
Yes								
No								
**Subcategorization of DSI**	0.004				< 0.001			
< full-thickness		Ref				Ref		
full-thickness		1.336	1.020 - 1.750	0.035		1.429	1.053 - 1.941	0.022
> full-thickness		1.737	0.907 - 3.327	0.096		1.803	0.870 - 3.735	0.113
**Tumor diameter (cm)**	0.117				0.005	1.593	1.182 - 2.184	0.002
≤ 4								
> 4								
**Lymphovascular space invasion**	< 0.001	1.826	1.415 - 2.356	< 0.001	< 0.001	2.240	1.672 - 3.000	< 0.001
Yes								
No								
**Recurrence region**	0.004				0.027			
Only pelvic								
Only extrapelvic								
Pelvic + extrapelvic								
**Adjuvant radiotherapy**	0.547				0.145			
Yes								
No								
**Adjuvant chemotherapy**	0.473				0.481			
Yes								
No								

## Abbreviations

DSI: deep stromal invasion
FIGO: International Federation of Gynaecology and Obstetrics
DFS: disease-free survival
OS: overall survival
LVSI: lymphovascular space invasion
NCCN: National Comprehensive Cancer Network
EBRT: external beam radiotherapy
