# Supplementary material for: The clinical and prognostic implication of deep stromal invasion in cervical cancer patients undergoing radical hysterectomy
Source: J Cancer. 2020 Oct 23;11(24):7368–77. doi: 10.7150/jca.50752 (PMC7646159; doi:10.7150/jca.50752)
Supplement: Supplementary file 1 — Supplementary tables. [file jcav11p7368s1.pdf]

Supplementary table.1 Multivariable analysis for DFS and OS in squamous carcinoma patients.

Variable	DFS Multivariate			OS Multivariate		
	HR	95% CI	P value	HR	95% CI	P value
**FIGO stage (2009)**	1.327	1.061 - 1.660	0.013	1.440	1.108 - 1.872	0.006
IB						
IIA						
SCCA (Continuous)	1.014	1.006 - 1.021	< 0.001	1.012	1.005 - 1.019	0.001
**Subcategorization of DSI**						
< full-thickness	Ref			Ref		
full-thickness	1.340	1.070 - 1.678	0.011	1.514	1.170 - 1.959	0.002
> full-thickness	1.908	1.361 - 2.676	< 0.001	1.898	1.290 - 2.793	0.001
**Tumor diameter (cm)**	1.159	0.940 - 1.430	0.167	1.239	0.977 - 1.571	0.077
≤ 4						
> 4						
**Lymphovascular space invasion**	1.924	1.516 - 2.442	< 0.001	2.233	1.686 - 2.958	< 0.001
Yes						
No						
**Lymph node metastasis**	1.567	1.242 - 1.977	< 0.001	1.512	1.158 - 1.976	0.002
Yes						
No						
**Parametrial involvement**	1.700	1.282 - 2.253	< 0.001	1.828	1.350 - 2.477	< 0.001
Yes						
No						
**Vaginal margin invasion**	1.675	1.142 - 2.455	0.008	1.650	1.085 - 2.510	0.019
Yes						
No						

Supplementary table.2 Multivariable analysis for DFS and OS in adenocarcinoma patients

**Variable**	DFS Multivariate			OS Multivariate		
	HR	95% CI	P value	HR	95% CI	P value
**FIGO stage (2009)**	1.180	0.677 - 2.057	0.559	1.212	0.670 - 2.192	0.525
IB						
IIA						
CA-125 (Continuous)	1.002	1.000 - 1.003	0.005	1.001	1.000 - 1.002	0.024
**Subcategorization of DSI**						
< full-thickness	Ref			Ref		
full-thickness	1.384	0.799 - 2.398	0.246	1.202	0.670 - 2.156	0.538
> full-thickness	2.181	0.895 - 5.314	0.086	1.738	0.716 - 4.220	0.222
**Tumor diameter (cm)**	1.446	0.859 - 2.435	0.165	1.345	0.781 - 2.317	0.285
≤ 4						
> 4						
**Lymphovascular space invasion**	1.544	0.899 - 2.652	0.116	1.532	0.860 - 2.726	0.147
Yes						
No						
**Lymph node metastasis**	2.759	1.541 - 4.940	0.001	3.189	1.683 - 6.044	< 0.001
Yes						
No						
**Parametrial involvement**	1.063	0.489 - 2.308	0.878	1.598	0.767 - 3.329	0.210
Yes						
No						
**Vaginal margin invasion**	1.947	0.511 - 7.424	0.329	1.606	0.337 - 7.646	0.552
Yes						
No						

Supplementary table.3 Multivariable analysis for DFS in adenosquamous carcinoma patients

**Variable**	DFS			OS		
	Multivariate			Multivariate		
	HR	95% CI	P value	HR	95% CI	P value
**FIGO stage (2009)**	1.966	0.742 - 5.208	0.174	1.668	0.575 - 4.836	0.346
IB						
IIA						
SCCA (Continuous)	1.028	0.976 - 1.083	0.302	1.048	0.981 - 1.120	0.161
CA-125 (Continuous)	1.001	0.999 - 1.003	0.452	1.002	1.000 - 1.005	0.035
**Subcategorization of DSI**						
< full-thickness	Ref			Ref		
full-thickness	0.853	0.312 - 2.327	0.756	1.017	0.339 - 3.055	0.976
> full-thickness	3.546	0.946 - 13.289	0.060	4.486	1.101 - 18.280	0.036
**Tumor diameter (cm)**	1.587	0.518 - 4.862	0.419	4.143	0.987 - 17.399	0.052
≤ 4						
> 4						
**Lymphovascular space invasion**	1.403	0.530 - 3.718	0.495	1.277	0.461 - 3.536	0.638
Yes						
No						
**Lymph node metastasis**	2.224	0.803 - 6.159	0.124	1.157	0.371 - 3.607	0.801
Yes						
No						
**Parametrial involvement**	1.153	0.138 - 9.622	0.895	1.331	0.155 - 11.439	0.794
Yes						
No						
**Vaginal margin invasion**	1.513	0.266 - 8.591	0.641	1.195	0.156 - 9.138	0.864
Yes						
No						
